# Research on the Mechanical Properties and Stretch Forming Simulation of Triaxial Geogrid with Different Pre-Punched Hole Diameters

**DOI:** 10.3390/polym14132594

**Published:** 2022-06-27

**Authors:** Xinbo Ren, Xinhai Zhao, Chao Zheng, Libin Song, Ji Liu, Zhiyuan Si

**Affiliations:** Key Laboratory for Liquid-Solid Structural Evolution and Processing of Materials, Ministry of Education, School of Materials Science & Engineering, Shandong University, 17923 Jingshi Road, Jinan 250061, China; 17853143923@163.com (X.R.); zhengchao@sdu.edu.cn (C.Z.); derby@sdu.edu.cn (L.S.); liuji9707@163.com (J.L.); 18860870173@163.com (Z.S.)

**Keywords:** triaxial geogrid, mechanical properties, numerical simulation, stretching process

## Abstract

In this paper, the tensile behavior of industrial polypropylene triaxial geogrid with different pre-punched hole diameters was studied by experiment and numerical analysis. The industrial polypropylene sheets with different diameters of circular holes were stretched at elevated temperature and then the tensile properties of triaxial geogrids at room temperature were evaluated. It was found that the pre-punched hole diameter of triaxial geogrid had a very close relationship with the mechanical properties. With the increase of the pre-punched hole diameter, the tensile strength of triaxial geogrid shows a trend of first increasing and then decreasing. Combined with numerical simulation, the optimal pre-punched hole diameter can be accurately obtained, and the distribution law of the width, thickness, stress, and strain of triaxial geogrid can be obtained. Under the condition of a stretching ratio of 3 and node spacing of 3 mm, it was found that the mechanical properties of industrial polypropylene triaxial geogrid was the best when the pre-punched hole diameter was 2.6 mm.

## 1. Introduction

High-strength geogrid is a very important geosynthetic material, which is increasingly used in the construction of steep slopes and in the reinforcement of bridge abutments [1]. The geogrid reinforced structure has strong bearing capacity, small deformation, and can maintain good mechanical properties for decades [2]. At present, the most widely used geogrid is plastic geogrid. Plastic geogrid is a polymer mesh with a certain pore structure obtained by stretching. The traditional plastic geogrids conclude uniaxial geogrids and biaxial geogrids. With the continuous improvement of engineering requirements, new multi-axial geogrids have been developed and put on the market. One of the most widely used multi-axial geogrids is triaxial geogrid.

**Figure 1:**
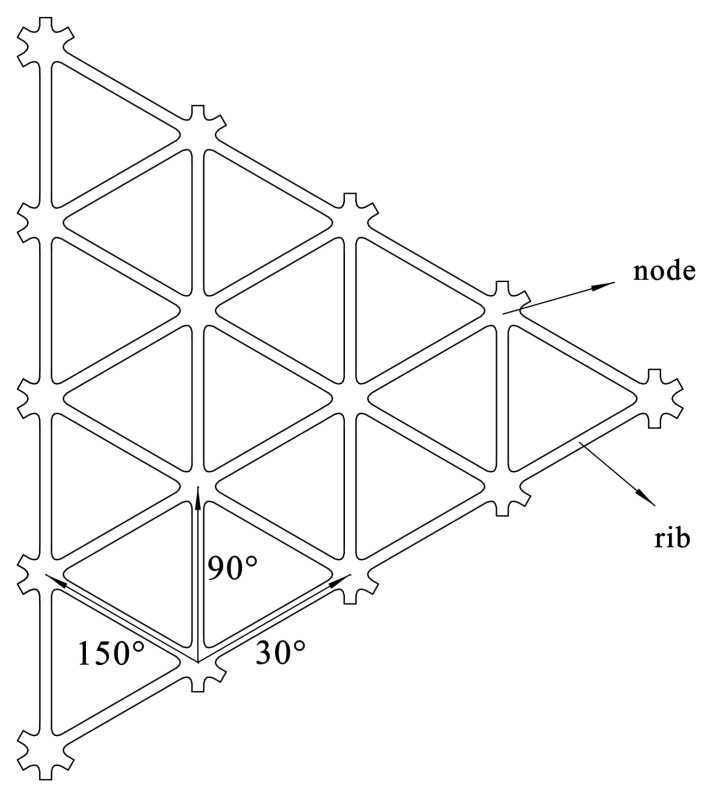
Basic structure of triaxial geogrid.

Triaxial geogrid is a new type of geogrid invented by Tensar [3]. Its forming process mainly includes sheet pre-punching, longitudinal, and transverse stretching at high temperature, cutting, and winding, etc. Its basic unit is generally an equilateral triangle, and its structure is shown in Figure 1. Taking the longitudinal stretching direction as the 90° direction, traxial geogrid is generally provided with ribs in the directions of 30°, 90°, and 150°. The 90° bars are called the longitudinal bars, and the others are named diagonal bars. Bars consist of nodes, ribs and transition areas. The nodes are basically not deformed during the forming process, while the ribs are the part mainly involved in the deformation, and the transition area connects the ribs and nodes.

Compared with a biaxial geogrid with ribs only at 0° and 90°, a triaxial geogrid has higher tensile strength in more directions. Through experiments, Dong et al. [4] found that the geogrid with triangular holes had a more stable structure and could bear more uniform tensile force in all directions, and used FLAC software to respond to the geogrid with rectangular and triangular holes under uniaxial tensile loads in different directions. The triangular hole geogrid was found to have a more uniform tensile strength and strength distribution than the rectangular hole geogrid. Zhang et al. [5] monitored the internal displacement along the length of the geogrid by burying the triaxial geogrid specimen in the compacted sand and conducting multiple pull-out tests. The research results showed that triaxial geogrid had more uniform tensile strength in each loading direction than the biaxial geogrid. Arulrajah et al. [6] found that the higher stiffness triaxial geogrid attained higher interface shear strength properties than that of the lower stiffness biaxial geogrid.

Triaxial geogrid can form an interlocking block with the soil and stone to improve the friction between the grid and the foundation soil, thereby improving the mechanical properties of the substrate. Das et al. [7] effectively increased the CBR (California Bearing Ratio) strength by reinforcing triaxial geogrid for rigid pavement. The California bearing ratio increased by 15% and 39% in the soaked condition when the Tx160 and Tx170 geogrids were interfaced in the sample, respectively. Ma et al. [8] used geogrid to reinforce the filling body in cut-and-fill sections, analyzed the force of the geogrid, established a tensile force calculation model, and obtained the theoretical calculation formula of the geogrid tensile force. Triaxial geogrid reinforcement was found to be more economical and effective in reducing differential settlement and lateral displacement of cut-and-fill embankments.

Therefore, triaxial geogrid is a new type of high-performance geosynthetic material with good application prospects. To improve the mechanical properties of triaxial geogrid, it is necessary to optimize the geogrid material, the process parameters of the pre-punched sheet, and the stretching process parameters.

The materials for producing a geogrid are generally high-density polyethylene (HDPE), industrial polypropylene (PP), and polyethylene terephthalate (PET). Among them, the material for producing triaxial geogrid is generally selected as industrial PP. In the industry, new PP materials and recycled materials are commonly mixed in a ratio of 2:1 as the basic material for the production of geogrids, which can reduce production costs on the premise of ensuring the stretching properties of PP [9]. Adding about 2% carbon black can inhibit the photoaging of the geogrid [10]. In addition, an appropriate amount of plasticizers and antioxidants should be added [11].

According to structure classification, PP can be divided into isotactic, syndiotactic, and atactic types [12]. Isotactic PP with high crystallinity is the main raw material for the production of various plastic products including geogrids. Isotactic PP contains two parts: a crystalline region and an amorphous region. The stretching process also includes the deformation of the first deformed amorphous region and the later deformed crystalline region [13]. Therefore, when plastic deformation occurs, PP tends to deform unevenly, resulting in “necking” [14].

Reinforcing materials can be added to polypropylene to improve certain properties. For example, experiments show that infusing PP with nanofibers increases the tensile modulus and yield strength, but decreases the ductility [15]. A modification method of the geogrid material is to add an appropriate amount of antioxidants to improve the service life of the geogrid. The antioxidants are effective to prevent polymer oxidation reaction in time [16], and the reaction rate of antioxidant depletion increased with temperature according to the Arrhenius equation, whereas the rate increased exponentially with oxygen pressure [17].

The process parameters of the pre-punched sheet determine the structure of the geogrid after stretching and have an important impact on the mechanical properties of the geogrid. Through stretch forming tests at elevated temperature and tensile fracture tests at room temperature, Zheng et al. [18] discovered the influence of the diameter of the circular pre-punching hole on the mechanical properties of the uniaxial geogrid. The transverse distance between circular pre-punched holes played important roles on the deformation and tensile fracture behavior of geogrids, and the too-small hole diameter limited the deformation of the rib and junction, resulting in forming failure of the geogrid. Ren et al. [19] found that under the condition of meeting the forming requirements, the smaller the diameter-to-distance ratio is beneficial to improve the tensile strength, nominal elongation, and performance utilization factor of the biaxial geogrid. When the longitudinal spacing is 1% larger than the transverse spacing, the material performance utilization factor is maximized.

The stretching process parameters, including stretching temperature, stretching speed, and stretching ratio, have a great effect on the tensile reinforcement of the geogrid [20]. The tensile temperature will affect the rheological properties of the material and the stress-strain distribution during the tensile process. Generally, the tensile temperature of industrial PP geogrids is 383–413 K. The experimental results show that the tensile stress-strain response of PP strongly depends on the applied strain rate and test temperature [21]. Generally speaking, decreasing the stretching speed and increasing the stretching temperature have the same effect on the stress-strain response [22]. The stretching ratio determines the size and shape of the geogrid. When the stretching ratio is too large, the ribs will be broken. On the contrary, if the stretching ratio is too small, it will lead to insufficient stretching and poor mechanical properties. Generally, the stretching ratio of multi-axial geogrid is about 3. In addition, the gap between punch and die will affect the accuracy of punching, thereby indirectly affecting the mechanical properties of the geogrid.

The stretching process at high temperature is the key to affect the performance of the geogrid. The geogrid tensile forming can be simulated and the forming results can be predicted by using Abaqus CAE software. This method is of great significance for obtaining the best pre-punching scheme for geogrids. At present, the numerical analysis of geogrid stretch forming is mainly limited to uniaxial and biaxial geogrids, and there are few simulations for triaxial or multi-axial geogrids. Caton-Rose et al. [23] used the elastic model of solid polymer with large deformation to predict the final shape of PP geogrids, verifying the feasibility of Abaqus simulation of uniaxial geogrids stretching process. Zheng et al. [18] studied the deformation behavior of isotactic PP in the manufacture of uniaxial geogrid by experimental and numerical methods. The effect of pre-punched holes on the mechanical properties of the uniaxial geogrid was obtained through the tensile test. It was found that the lateral spacing of holes had a great influence on the forming performance and fracture behavior of the grid. Ren et al. [19] optimized the basic tensile simulation element of biaxial geogrid, established a “biaxial tensile model” and used Abaqus software to simulate the biaxial geogrid tensile forming process, which could obtain accurate results of the forming regulation.

Therefore, combined with the above research, the authors speculate that the diameter of the pre-punched hole will have a very important impact on the mechanical properties of triaxial geogrid. Through the forming and tensile failure test combined with the numerical simulation of tensile forming, the authors will study the mechanical properties of triaxial geogrid with different diameters of the pre-punched holes, obtain the relationship between the mechanical properties of the triaxial geogrid and the diameter of the pre-punched holes, and further obtain the optimal pre-punched diameter of a triaxial geogrid. This research is of great help to improve the application value of a triaxial geogrid.

## 2. Stretch Forming and Tensile Fracture Tests of Triaxial Geogrid

In this chapter, the effect of pre-punched hole diameter on the mechanical properties of a triaxial geogrid will be studied through experiments. Firstly, the process parameters of pre-punched sheet and stretching will be designed. Then, the stretching forming test will be carried out, and the forming properties of a geogrid with different pre-punched holes will be compared. Finally, the mechanical properties of triaxial geogrid after forming will be tested at room temperature, and the mechanical properties of the geogrid will be compared.

### 2.1. Experimental Design

In this section, the process parameters of pre-punched sheet and stretching are designed, and the mechanical property parameters of the tensile fracture test are defined.

#### 2.1.1. Design of the Process Parameters of the Pre-Punched Sheet and Stretching

The industrial PP sheets used in this paper were provided by Feicheng Lianyi Engineering Plastics Co., Ltd., Taian, Shandong, China, and the thickness of the sheets was 4 mm. Because the round punch is simple to process and easy to debug and maintain, in this paper, the pre-punched holes of the industrial PP triaxial geogrid are all circular.

**Figure 2:**
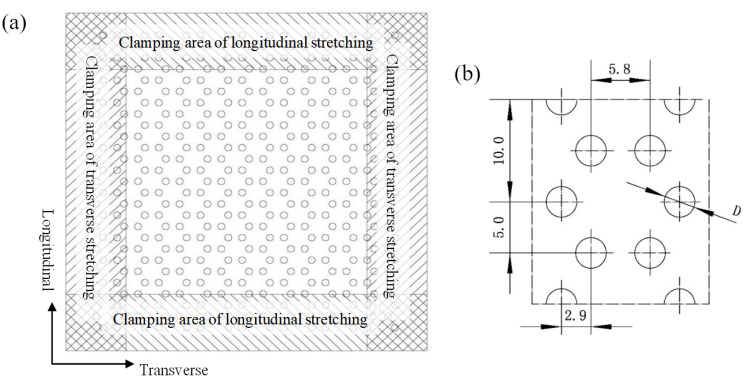
Pre-punched sheet of a triaxial geogrid. (**a**) Clamping area of pre-punched sheet, (**b**) process parameter diagram of the structure of the pre-punched sheet.

The first step is to punch the industrial PP sheet. In order to ensure that the triaxial geogrid sheet after high temperature stretching has enough uniform deformation area, the pre-punched sheet should be large enough. In this test, the pre-punched sheet is square, with a length and width of 150 mm, leaving a clamping area of 25 mm on each side, as shown in Figure 2a. The process parameters of the pre-punched structure are shown in Figure 2b. The diameter of the circular pre-punched hole (D) is a variable, taking the values 2.0, 2.5, 3.0, 3.5, and 4.0, and the unit of it is mm.

Then, stretch forming tests are performed on the pre-punched sheets. The optimal temperature and stretching speed of using industrial PP material to produce the geogrid are 393 K and 100 mm/min through experiments [22]. The pre-punched sheets are stretched longitudinally at high temperature. The holding time at elevated temperature is 5 min, the stretching temperature is 393 K, and the stretching speed is 100 mm/min. Subsequently, the pre-punched sheets are stretched transversely under the same process parameters.

Compared with biaxial geogrid, the pre-punched structure of multi-axial geogrid is more complicated, and the general stretching ratio should be lower. In this test, the stretching ratio of the triaxial geogrid is 3, and the distance between adjacent nodes is 30 mm. The stretching process parameters are shown in Table 1.

#### 2.1.2. Mechanical Properties Index of the Tensile Fracture Test

The mechanical properties and product naming rules of uniaxial and biaxial geogrids are specified in Chinese national standard GB/T 17689-2008 [24] and American national standard ASTM D6637 [25]. The color of plastic geogrids should be uniformly black; the appearance should be free of damage or cracks, the mesh size and shape should be uniform, and the carbon black content should not be less than 2%. The relevant mechanical performance indicators include tensile strength, nominal tensile strength, nominal elongation, and creep, etc. The single-rib method or the multi-rib method can be used to test the mechanical properties of the geogrid.

Compared with uniaxial and biaxial stretched geogrids, multi-axial geogrids currently lack the corresponding relationship between relevant product specifications and specific parameters. According to the characteristics of the multi-axial geogrid, tensile strength and elongation at break are selected as the mechanical performance indicators of the multi-axial geogrid. Furthermore, the concept of multi-axial average tensile strength is proposed by the authors.

**Figure 3:**
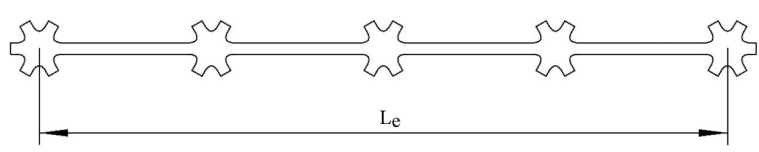
Sampling method for the tensile test at room temperature.

The multi-axial geogrid was sampled by the single-rib method at room temperature, and the samples were subjected to room temperature tensile fracture test by electronic universal testing machine. The part with nodes and ribs evenly distributed and flat was selected as sample. The length of the sample should include at least two basic elements, and the effective length should not be less than 100 mm. The sampling method is shown in Figure 3.

In Figure 3, $L_{e}$ is the effective length of the sampling, which represents the distance between the center positions of the nodes at both ends of the sampling. In this test, $L_{e}$ should be greater than 100 mm. During the clamping process, it should be ensured that the clamps clamp the node parts at both ends of the sample, and make sure that the tensile direction is parallel to the holding direction of the sample.

The tensile strength, elongation at break and multi-axial average tensile strength of the samples were obtained through the tensile fracture test at room temperature. The definitions and calculation methods are as follows.

(1) Tensile strength is one of the most important indicators to evaluate the performance of a geogrid, and its calculation method is shown in Equation (1).

(1) $$F=\frac{fN}{nL_{e}}$$

*F* is the tensile strength, which unit is kN/m. *f* is the tensile force value of the sample, and the unit is kN. *N* is the number of ribs in the direction of the sample to be measured. *n* is the number of ribs in the non-measured direction. *n* is no less than 2 in multi-rib method, and equals to 1 in single-rib method. $L_{e}$ is the effective width of the sample in the direction to be measured, in m.

(2) The elongation at break represents the ductility of the geogrid under tension, and its calculation method is shown in Equation (2).

(2) $$\delta =\frac{\Delta G}{G_{0}}$$

Geogrids with large elongation at break are prone to node offset under the action of tensile force, which changes the appearance of the product and leads to failure. Therefore, the elongation at break should not be too large. In Equation (2), δ represents the elongation at break of the geogrid; ΔG represents the displacement of the fixture along the tensile direction when it breaks in mm; $G_{0}$ represents the distance between the fixtures under the pre-tension state.

(3) The multi-axial average tensile strength can be used to compare the average tensile strength of the multi-axial geogrid in all directions, and then measure the comprehensive mechanical properties of the multi-axial geogrid. The calculation method is shown in Equation (3).

(3) $$\bar{F}=\frac{\sum_{i=1}^{s}F_{i}}{s}$$

If a basic unit of a geogrid contains ribs, the tensile strength in the direction of each rib $F_{1},F_{2},F_{3},\ldots ,F_{s}$ needs to be obtained first, and then the average of the tensile strength of all ribs is calculated.

### 2.2. Stretch Forming Tests of Triaxial Geogrid

**Figure 4:**
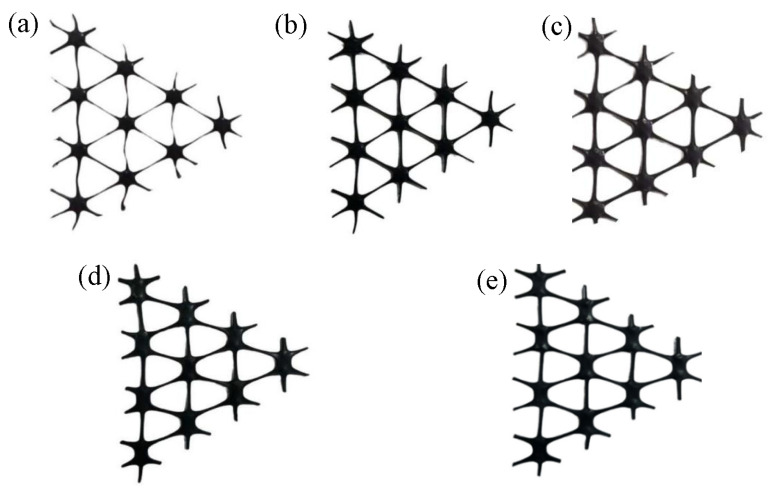
Circular pre-punched industrial PP triaxial geogrid with different diameters. (**a**) D = 4.0 mm, (**b**) D = 3.5 mm, (**c**) D = 3.0 mm, (**d**) D = 2.5 mm, (**e**) D = 2.5 mm.

Stretch forming tests were performed on the pre-punched sheets shown in Figure 2 according to the process parameters in Table 1. Figure 4 shows the uniformly deformed part of the center of the triaxial geogrid with different pre-punched hole diameters.

**Figure 5:**
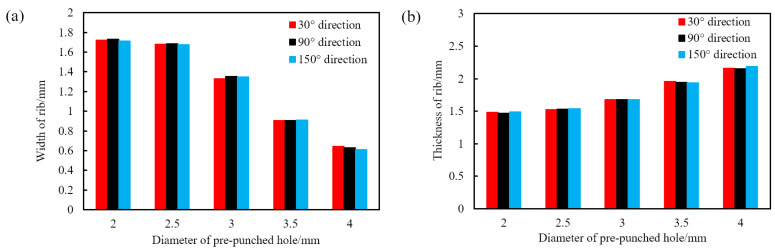
Rib width and thickness of an industrial PP triaxial geogrid with different pre-punched holes. (**a**) Width, (**b**) thickness.

As shown in Figure 4, the rib width of the triaxial geogrid gradually decreases and the nodes gradually become smaller with the increase of the diameter of the pre-punched hole. When D = 4.0 mm, the longitudinal ribs are bending, while this will not happen when D is less than 4.0 mm. We measure the width and thickness of the ribs for triaxial geogrids with different pre-punched holes after stretch forming, as shown in Figure 5.

As shown in Figure 5, with the increase of the diameter of the pre-punched holes, the width of the triaxial geogrid rib gradually decreases, and the thickness gradually increases. The width and thickness of the rib are closest to each other when D = 2.5 mm. When D is not less than 3.0 mm, the width is less than the thickness. When D = 2.0 mm or 2.5 mm, the width is greater than the thickness. When D = 4.0 mm, 3.5 mm, or 3.0 mm, the middle part of the node of the triaxial geogrid is convex, and the thickness gradually decreases in all directions. When D = 2.0 mm, plastic deformation occurs in the central area of the node, resulting in a depression, and the thickness of the part near the rib is larger. When D = 2.5 mm, there are two kinds of nodes at the same time.

**Figure 6:**
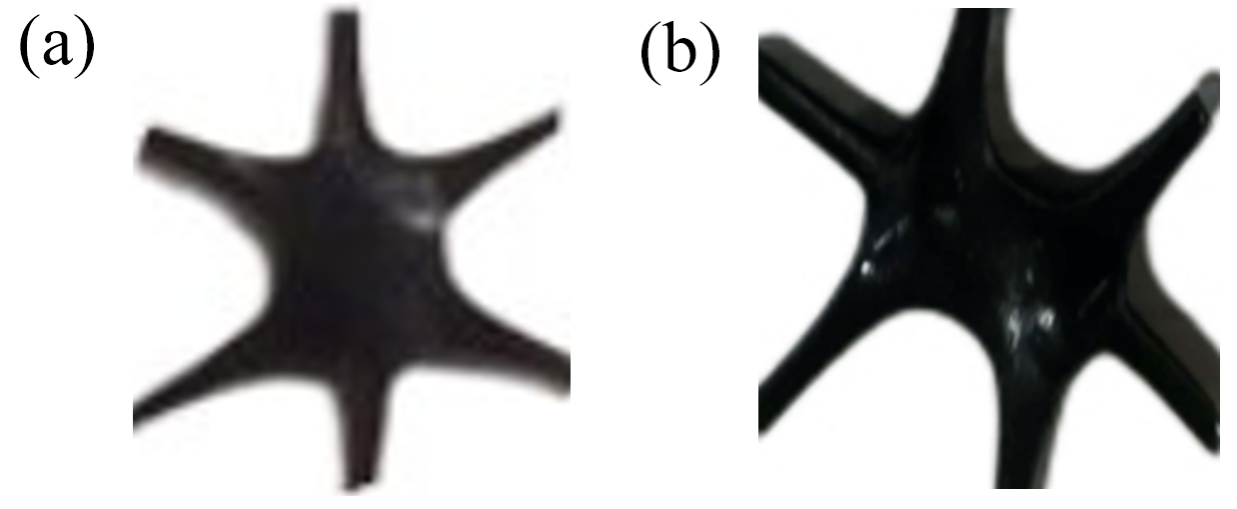
Nodes of different shapes. (**a**) Convex nodes in the middle, (**b**) concave nodes in the middle.

The above-mentioned nodes can be divided into two categories, namely, concave nodes in the middle and convex nodes in the middle, as shown in Figure 6. It was observed that in the process of high temperature stretching, the first deformation region was dominated by the concave nodes in the middle, and the post deformation region was dominated by the convex nodes in the middle.

The reason for the generation of concave nodes in the middle is that during the longitudinal stretching process, due to the small size of the pre-punched holes and the large width of the longitudinal ribs, necking is difficult to occur, so the phenomenon of necking of nodes may occur. Therefore, convex nodes in the middle will be created. Due to the deformation of the nodes, the ribs in the 30° direction under the conditions of D = 2.5 mm and D = 2.0 mm are not on the same line, but the ribs remain parallel, and the ribs in the 150° direction show the same change.

### 2.3. Tensile Fracture Tests at Room Temperature of Triaxial Geogrid

**Figure 7:**
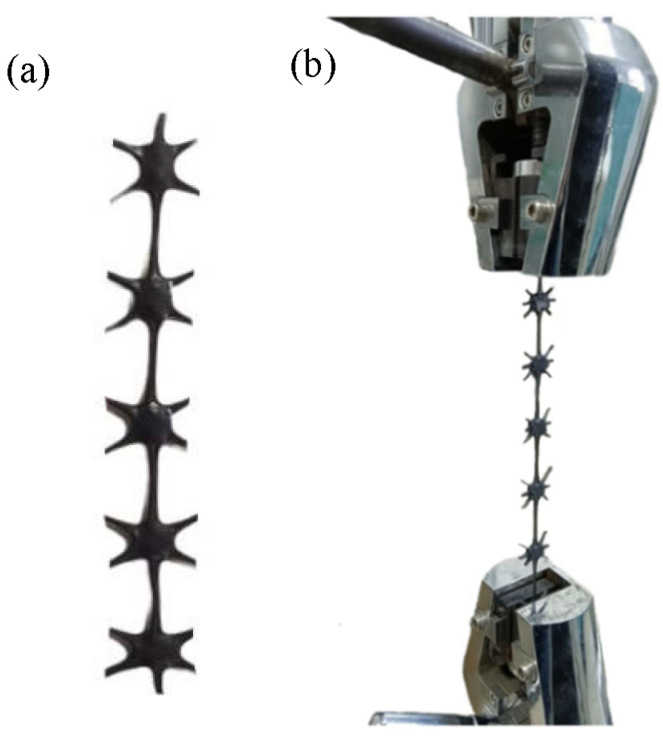
Test method for the mechanical properties of single rib method. (**a**) Selection of samples, (**b**) clamping method.

In the tensile fracture test at room temperature, the triaxial geogrids obtained after stretch forming were sampled to obtain samples with straight ribs and evenly distributed nodes. The length of the samples was not less than 100 mm. Then the mechanical properties were tested using an electronic universal testing machine, and the tensile strength, elongation at break and multi-directional average tensile strength were obtained. Selection of samples and clamping method are shown in Figure 7.

**Figure 8:**
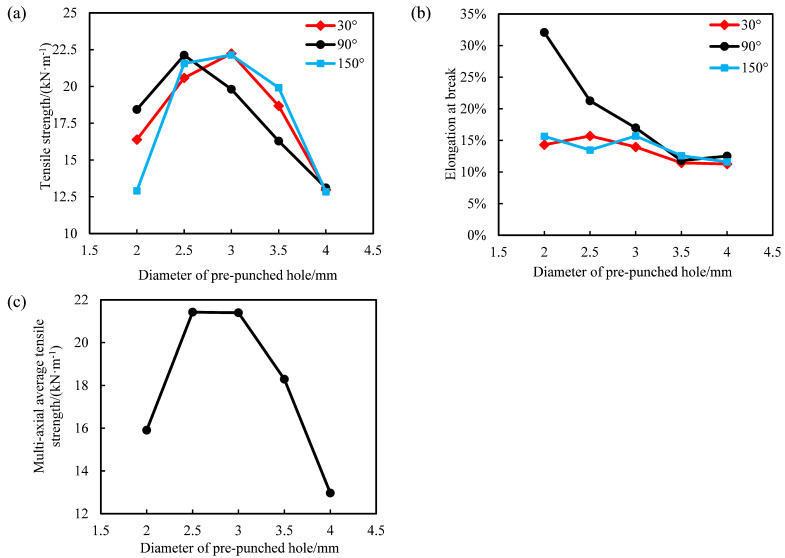
The relationship between the mechanical properties and the diameter of the pre-punched holes of triaxial geogrid. (**a**) Tensile strength, (**b**) elongation at break, (**c**) multi-axial average tensile strength.

In this test, we take 20% of the distance between the clamps as the tensile speed per minute [24]. At least four experimental data of each group were taken for analysis. The relationship between the mechanical properties and the diameter of the pre-punched holes of the triaxial geogrid is shown in Figure 8.

It can be seen from Figure 8a that with the increase of the diameter of the pre-punched hole, the tensile strength in each direction increases first and then decreases. The tensile strength in the 30° and 150° directions reaches the maximum when D = 3.0 mm, while the tensile strength in the 90° direction takes the maximum value when D = 2.5 mm. The tensile strength in all directions showed a trend of first increasing and then decreasing. When D = 4.0 mm, the tensile strength in all directions was low. The main reasons for the above phenomenon are as follows.

(1) When D = 2.0 mm, the plastic deformation of the node occurs during the tensile process, the integrity of the node is damaged, the tensile strength of the node is low, and the fracture occurs at the node during the tensile fracture test, resulting in a lower tensile strength of the spline under this condition.

(2) When D = 2.5 mm, the phenomenon of plastic deformation of the node occurs, but due to the low degree, and the large width and thickness of the rib, the tensile strength of ribs in each direction is high.

(3) When D = 3.0 mm, the joint structure is complete, no plastic deformation occurs, and the overall tensile strength is high.

(4) When D = 3.5 mm or 4.0 mm, because the pre-punched hole is too large, there is less material to form the rib during the stretch forming process, leading to the small width of the rib after forming. In addition, the ribs are partly bent with a pre-punched diameter of 4.0 mm. Therefore, the tensile strength in all directions is low under this condition.

It can be seen from Figure 8b that the elongation at break in the 90° direction of the triaxial geogrid shows a decreasing trend with the increase of the diameter of the pre-punched hole. The elongation at break in the 30° and 150° directions has little relationship with the diameter of the pre-punched hole, and the elongation at break is generally 10% to 15%. This is because when D equals to 2.0 mm or 2.5 mm, the nodes undergo plastic deformation along the 90° direction during longitudinal stretching, and the middle of the nodes is concave during this process. In the process of breaking at room temperature, the concave nodes continued to thin and were completely pulled apart, and some splines appeared fractured in the node, which greatly increased the fracture elongation. When D = 3.0 mm, 3.5 mm or 4.0 mm, the elongation at break of the rib in the three directions is basically equal. During the room temperature tensile fracture test, the fracture area is the transition zone or rib.

It can be seen from Figure 8c that with the increase of the diameter of the pre-punched hole, the multi-axial average tensile strength of triaxial geogrid first increases and then decreases; when D = 2.5 or 3.0 mm, the multi-directional average tensile strength of triaxial geogrid reaches the maximum value, which is about 21 kN·m${}^{-1}$.

In order to keep the shape of the geogrid stable under load, the elongation at break of plastic tensile geogrid should not be too large [24]. Compared with triaxial geogrid with D = 2.5 mm, the elongation at break is lower when D = 3.0 mm, so the comprehensive performance of triaxial geogrid under this condition is better. Due to the large sampling interval, the maximum value of the multi-axial average tensile strength is located in the sampling interval of (2.5 mm, 3.0 mm). Therefore, when D is in the range of 2.5 mm to 3.0 mm, the comprehensive mechanical properties of industrial PP triaxial geogrid are the best.

Therefore, when the stretching ratio is 3 and the distance between each node is 30 mm, the optimal pre-punched diameter of industrial PP triaxial geogrid is between 2.5 and 3.0 mm.

According to the experiments on biaxial geogrids of the same material by Ren et al. [19], the maximum tensile strength of biaxial geogrids is 23 kN·m${}^{-1}$, which is slightly larger than the triaxial geogrid. However, the triaxial geogrid can carry loads in more directions, and has a stable structure and an obvious interlocking effect with soil and stone, so it has better application prospects.

In this chapter, stretch forming tests at elevated temperature and tensile fracture tests at room temperature were carried out on the triaxial geogrid with different pre-punched hole diameters, and the triaxial geogrid was analyzed from four angles: tensile strength, elongation at break, multi-axial average tensile strength, and comparison with the biaxial geogrid. The relationship between the mechanical performance of triaxial geogrid and the diameter of the pre-punched hole was analyzed. Meanwhile, the optimal range of the pre-punched diameter of the triaxial geogrid was obtained, and the reasons were analyzed.

## 3. Simulation Research on the Forming of Triaxial Geogrid

In Section 2, the authors studied the mechanical properties of the industrial PP triaxial geogrid by means of experiments. However, it is difficult to study the concrete forming process of the triaxial geogrid and the distribution law of stress and strain by means of experiments. In addition, the maximum value of the multi-axial average tensile strength obtained from the test is in the range of 2.5 mm to 3.0 mm. However, due to the large sampling interval, the specific value cannot be obtained. By means of numerical simulation analysis, the maximum point can be obtained simply and quickly without experiments, saving a lot of time and material resources. In this chapter, the Abaqus software is used to simulate and analyze the heat transfer and tensile forming process of triaxial geogrid, and the thickness, width, stress and strain distribution of the triaxial geogrid with different pre-punched diameters are obtained.

### 3.1. Establishment of Triaxial Geogrid Constitutive Model

Algorithms in Abaqus CAE include the explicit algorithm and implicit algorithm. Compared with the implicit algorithm, the explicit algorithm is more suitable for dynamic analysis, as there is no need for equilibrium iteration, and the calculation speed is faster [26,27,28]. Therefore, the explicit algorithm is suitable for the numerical simulation analysis of geogrid stretch forming.

The material models in Abaqus CAE conclude linear elasticity, elasto-plasticity, hyperelasticity, hypoelasticity, hyperelastic foam, viscoelasticity, etc. [29]. Among them, linear elasticity is the simplest material model in Abaqus CAE, which can define isotropic, orthotropic, anisotropic, and other material behaviors, and is suitable for small elastic strains. The elasto-plasticity model must meet the small deformation conditions, that is, the displacement of each point inside the object is much smaller than the original size of the object. The hyperelasticity model can be used to describe an almost incompressible model, which is suitable for large deformations, especially large volume changes, and the mechanism of permanent deformation is added (hyperelasticity with permanent set), which can numerically analyze the stretching process of polymers in various glassy or superelastic states. The Marlow constitutive model in the hyperelastic model can accurately fit the stress-strain curve with a yield point, and accurately simulate the yield-necking-hardening process of glassy polymers.

The Marlow constitutive model uses strain potential energy to describe the stress-strain relationship of hyperelastic materials. The strain potential energy defines the stored strain energy per unit volume of a material as a function of the strain at that point. The strain potential energy function of the material in the Marlow model can be expressed as Equation (4).

(4) $$U=U_{dev}\left(\bar{I_{1}}\right)+U_{vol}\left(J_{el}\right)$$

In Equation (4), *U* is the unit strain potential energy, $U_{dev}$ is the stress-strain potential energy, $U_{vol}$ is the volumetric strain potential energy, $J_{el}$ is the elastic volume ratio, and $\bar{I_{1}}$ is the first-order deviatoric strain invariant, which can be expressed by Equation (5).

(5) $$\bar{I_{1}}={\bar{\lambda_{1}}}^{2}+{\bar{\lambda_{2}}}^{2}+{\bar{\lambda_{3}}}^{2}$$

In Equation (5), $\bar{\lambda_{i}}=J^{-1/3}\lambda_{i}$, *J* is the total volume change rate and $\bar{\lambda_{i}}$ is the draw ratio in the main direction. In Equation (4), the stress part energy is determined by the uniaxial, biaxial, or planar test data, and the volume part energy is determined by the volume test data [19].

Therefore, it is necessary to carry out tensile tests on industrial PP materials, obtain stress-strain curves and import them into Abaqus software, in order to simulate the tensile forming process of triaxial geogrids.

**Figure 9:**
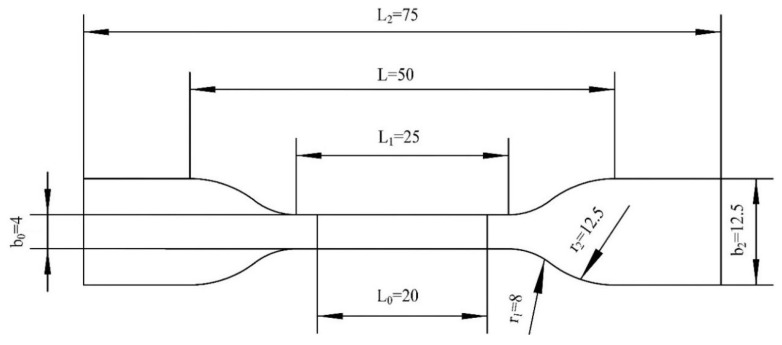
Schematic diagram of 5A standard sample.

According to Chinese national standard GB/T 1040.2-2006 [30], considering the limitation of the test equipment travel, it is advisable to use the 5A standard sample to sample industrial PP, as shown in Figure 9.

In Figure 9, $L_{0}$ is the gauge length of the 5A spline, $L_{1}$ is the length of the narrow parallel part, $L_{2}$ represents the total length of the 5A spline, and *L* is the initial distance between the fixtures. $b_{0}$ represents the width of the narrow part, and $b_{2}$ is the length of the spline end. $r_{1}$ and $r_{2}$ represent the small and large radii of the transition zone of the end and parallel sections respectively.

**Figure 10:**
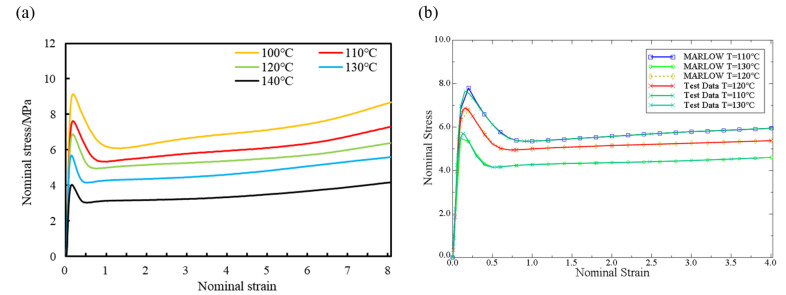
Nominal stress-strain curves of industrial PP. (**a**) Experimental value, (**b**) Fitting of the Marlow curve.

Industrial PP sheets were sampled and tested at high temperature. The tensile rate was set at 100 mm/min and the stretching ratio was set at 8 times, and the nominal stress-strain curves of the material were obtained at 373 K, 383 K, 393 K, 403 K, and 413 K, as shown in Figure 10a.

Abaqus CAE obtains the material strain potential energy function through the material stress-strain data using the least squares method [29]. The simulation assumes that industrial PP is an isotropic material, and the stress-strain data is obtained through uniaxial tensile tests and imported into the analysis. In this numerical simulation, the 383–403 K stress-strain curve of industrial PP is used for fitting, as shown in Figure 10b.

It can be seen from Figure 10a that under various temperature conditions in the range of 373–413 K, the nominal stress increases with increasing nominal strain before the yield point and first increases and then decreases after yielding. With the increase of temperature, the yield stress of industrial PP gradually decreased, the corresponding yield strength showed a slightly decreasing trend, and the elastic modulus also decreased gradually with the increase of temperature. Under the same nominal strain condition, the higher the temperature, the lower the nominal stress of the industrial PP material. The polymer has good tensile properties at high temperature, and remain unbroken under the condition of a larger stretching ratio.

It can be seen from Figure 10b that in the elastic section, there is a small deviation between the fitting of the Marlow curve and the experimental value. After reaching the yield point, the fitting of the Marlow curve is basically consistent with the experimental value. Since the stretching process is mainly the deformation of the plastic section, the deformation can basically be completely retained after removing the external force. The Marlow curve can better describe the stress-strain behavior of industrial PP during the stretching process at 383–403 K, so in the numerical simulation part, the Marlow constitutive model is selected.

**Figure 11:**
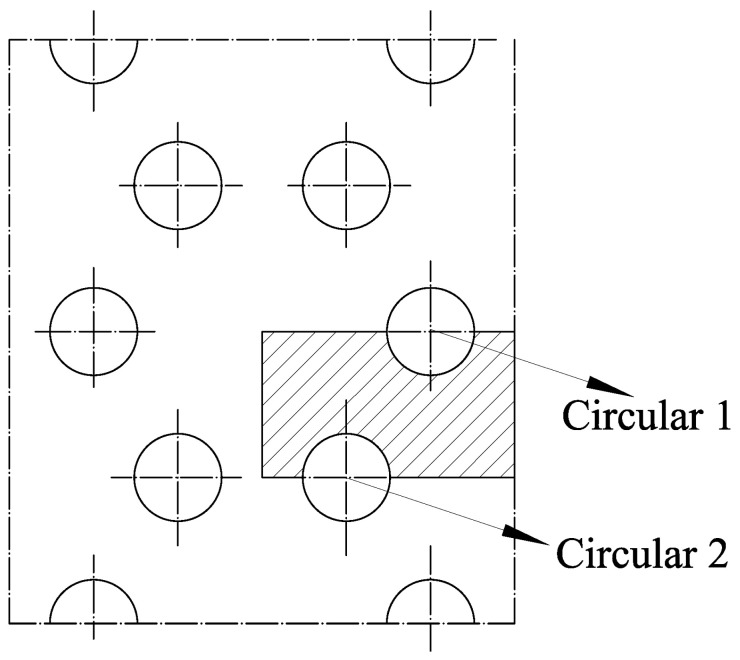
Simulation unit of triaxial geogrid.

The method of appropriately reducing the size of the model can improve the simulation speed due to the symmetry of pre-punched plate [31]. For the triaxial geogrid, the selection of the basic simulation element from the pre-punched sheet is shown in the shaded area in Figure 11.

The triaxial geogrid simulation unit has a length of 8.7 mm, a width of 5 mm, and a thickness of 4 mm. In Figure 11, the distance between the center of Circular 1 and the right boundary of the simulation unit is 2.9 mm, and the distance between the center of Circular 2 and the left boundary of the simulation unit is 2.9 mm. Once the basic elements of the simulation are set up, heat transfer simulations, and biaxial stretching simulations can be performed.

**Figure 12:**
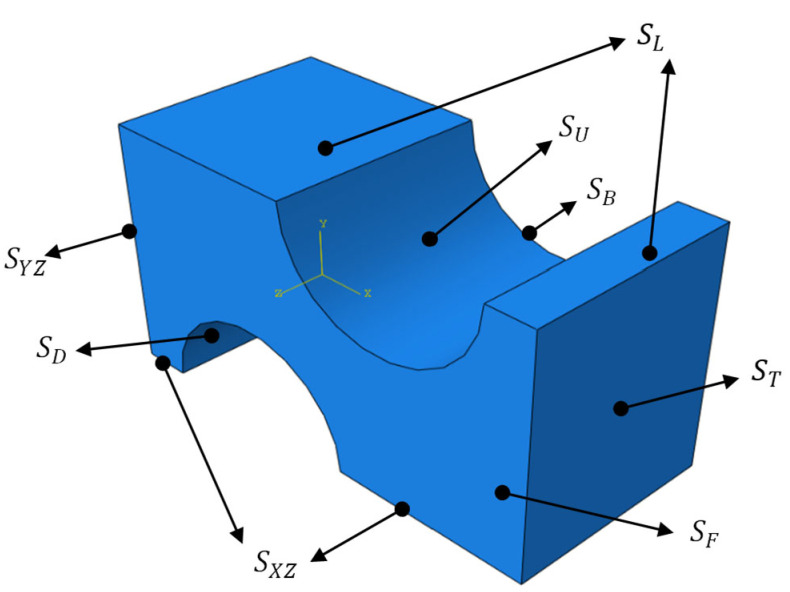
Loading surface and symmetry plane of the basic simulation unit.

We model the basic simulation unit and name the loading surface as shown in Figure 12.

In Figure 12, $S_{U}$ and $S_{D}$ are the punching surfaces. $S_{F}$ and $S_{B}$ are the front and behind surfaces. $S_{YZ}$ and $S_{XZ}$ are the symmetry planes of the model along the XZ surface and the YZ surface. $S_{L}$ and $S_{T}$ are the loading surfaces of longitudinal stretching and transverse stretching.

### 3.2. Heat Transfer Simulation of Industrial PP Triaxial Geogrid

Before the industrial PP triaxial geogrid is stretched longitudinally, the pre-punched sheet needs to be heated at a certain temperature. In this section, the heat transfer simulation of the heating process of industrial PP triaxial geogrid pre-punched sheets with different pre-punched diameters is carried out, and the relationship between the temperature distribution during the heating process and the diameter of the pre-punched holes is obtained.

The heat transfer process includes step Initial for describe initial conditions and Step-1 for describing the heat transfer process. In Initial, we set the model to be mirror-symmetrical along $S_{YZ}$ and $S_{XZ}$, and inherit the boundary conditions into Step-1. In Step-1, we set the interaction conditions to simulate the heat transfer between the model and the hot air in the oven, and $S_{F}$, $S_{B}$, $S_{U}$, and $S_{D}$ are the heat transfer surfaces. In this numerical simulation, the thermal conductivity of industrial PP is set to 0.24 [32], the temperature before heat preservation is 293 K, the target temperature is 393 K, and the high temperature holding time is 5 min.

The mesh type is set to the eight-node linear heat transfer hexahedron element DC3D8, and the mesh size is about 0.15 mm. We create a new task and submit it for analysis [33,34,35].

**Figure 13:**
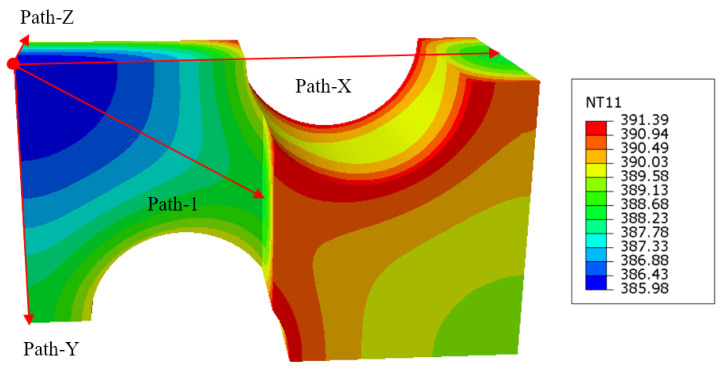
Heat transfer results and path specification of triaxial geogrid with a pre-punched diameter of 3.5 mm.

Taking the triaxial geogrid with a pre-punched hole diameter of 3.5 mm as an example, the heat transfer results and the setting of the path are shown in Figure 13.

It can be seen from Figure 13 that after the heat transfer, the temperature at different positions is different. The temperature in the area close to the heat transfer surfaces is higher, and this part is easy to deform first in the stretching process, thereby forming ribs. The core material is far away from the heat transfer surface, the heat transfer is insufficient, and the temperature is lower after the heat transfer, which makes it easy to form nodes in the subsequent stretching process. The temperature at the junction of the circular pre-punched holes and the surface of the sheet is the highest after heat transfer, up to 391 K, and the lowest temperature of the core material is 386 K.

**Figure 14:**
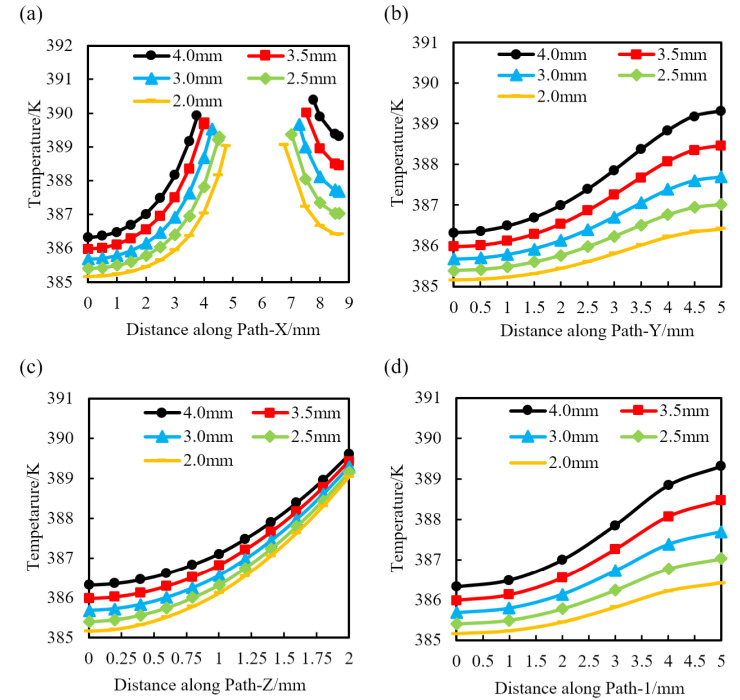
Temperature distribution of triaxial geogrid with a different pre-punched diameter along each path. (**a**) Path-X, **(b)** Path-Y, (**c**) Path-Z, (**d**) Path-1.

Heat transfer simulations were performed on triaxial geogrids with different pre-punched diameters, and the temperatures along Path-X, Path-Y, Path-Z, and Path-1 were calculated, as shown in Figure 14.

It can be seen from Figure 14a that the pre-punched holes will be passed along Path-X. Before Path-X passed the pre-punched hole, the temperature gradually increases along Path-X, and, after Path-X passed the pre-punched hole, the temperature gradually decreases. For samples with the same pre-punched diameter, the temperature after Path-X passed the pre-punched hole is slightly higher than that before Path-X passed the hole.

It can be seen from Figure 14b that the temperature of each sample along Path-Y gradually increases, and the temperature difference at the end of Path-Y with different pre-punched diameters is greater than the temperature difference at the starting point of Path-Y.

It can be seen from Figure 14c that the temperature of each sample along Path-Z gradually increases, and the temperature difference at the end of Path-Z for samples with different pre-punched diameters is smaller than the temperature difference at the starting point of Path-Z.

It can be seen from Figure 14d that the temperature distribution law of the sample along Path-1 direction is basically consistent with that along Path-Y, showing a trend of increasing temperature along Path-1. Furthermore, the temperature difference at the end of Path-1 is greater than that at the beginning.

Therefore, Figure 14 shows that with the increase of the diameter of the pre-punched hole, the temperature of the same position on each path increases continuously, indicating that the larger the pre-punched hole is, the better the heat transfer effect is. At the same time, the temperature of each part is different under the same pre-punched condition, indicating that the subsequent stretching process is carried out under a certain temperature gradient, and an appropriate temperature gradient is beneficial to the forming of the geogrid.

### 3.3. Simulation of Stretch Forming of Industrial PP Triaxial Geogrid

In this section, stretch forming simulation of the geogrid will be performed. The stretching process of the triaxial geogrid includes longitudinal stretching and transverse stretching in turn.

In the Initial analysis step, we set the heat transfer simulation result file to a predefined field. We set the "Dynamic, Temperature-Displacement, Explicit” analysis step, Step-1, to represent longitudinal stretching, and Step-2 to represent transverse stretching.

In step Initial, we set the model to be mirror-symmetrical along $S_{XZ}$ and $S_{YZ}$, and inherit the boundary conditions in the Step-1 and Step-2 analysis steps. In Step-1, a load is applied to $S_{L}$ so that $S_{L}$ is displaced by 10 mm in the direction of longitudinal stretching, and the model is constrained to be mirror-symmetrical along $S_{T}$. In Step-2, the boundary condition set in Step-1 is modified to “inactive”, and a load is applied to $S_{T}$, so that $S_{T}$ is displaced 17.3 mm in the direction of transverse stretching, and the model is constrained to be mirror-symmetrical along the $S_{L}$. This ensures that the longitudinal and transverse stretch ratios are both 3.

After the loads and boundary conditions are set, the model needs to be meshed. In this numerical analysis, the eight-node thermally coupled hexahedral element C3D8T is used. The mesh size is 0.15 mm, which is consistent with the heat transfer step. After meshing the model, we create a new job and submit it for analysis.

#### 3.3.1. Shape Analysis of Stretch Forming

**Figure 15:**
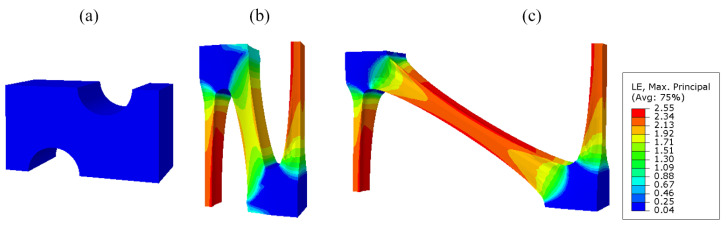
Strain cloud map of the triaxial geogrid stretch forming simulation. (**a**) Before longitudinal stretching, (**b**) after longitudinal stretching, (**c**) after transverse stretching.

Taking the industrial PP triaxial geogrid with a diameter of 3.5 mm as an example, the simulated stretch forming process is shown in Figure 15.

As shown in Figure 15, the node is basically not deformed during the stretching process, and the main deformation area is the rib. During the longitudinal stretching process, the deformation degree of the 90° bars is greater than that of the diagonal bars. During the longitudinal stretching process, the transition zone is basically formed. The transverse stretching process is mainly the stretching process in which the diagonal ribs are deformed in the transverse direction.

In addition, it was found that during the stretching process, the 90° rib shrinks inwards in both the width and thickness directions, and the rib is thin in the middle and thick on both sides. This is because during the stretching process, the rib is subjected to inward compressive stress, and the edge and corner areas are prone to stress concentration and are not easily deformed, so the shape is thin in the middle and thick on both sides. During transverse stretching, the 90° rib will decrease in thickness and increase in width.

Furthermore, after longitudinal stretching, the cross-sectional area of the 90° rib is smaller than before stretching. This is due to the fact that uniaxial stretching and equal biaxial compression are equivalent for a nearly incompressible material such as industrial polypropylene. During longitudinal tension, the cross-sectional area of the 90° rib is continuously reduced.

**Figure 16:**
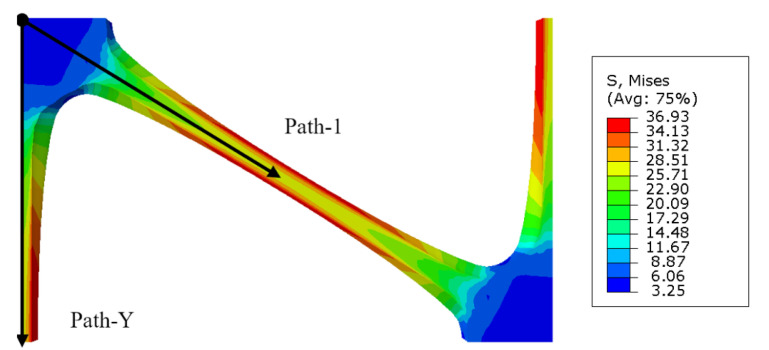
Cross section of the thick symmetry axis and path definition of triaxial geogrid.

The cross-section of the triaxial geogrid after transverse stretching is taken along the axis of symmetry in the thickness direction, as shown in Figure 16.

It can be seen from Figure 16 that the maximum stress and strain of a triaxial geogrid after stretching appears on the surface of the rib. Paths are defined along the longitudinal and diagonal directions, named Path-Y and Path-1. Due to the symmetry of the sample, in order to shorten the analysis time, the starting point is selected as the center of the node, and the end point is selected as the center of the longitudinal and diagonal ribs.

**Figure 17:**
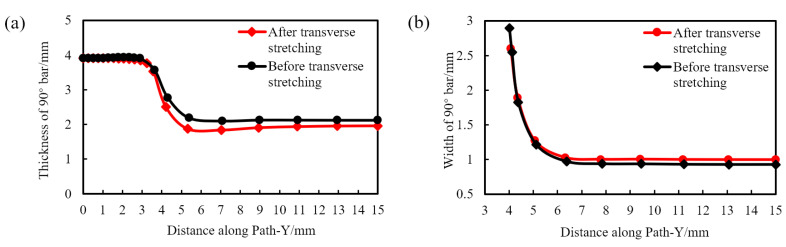
Thickness and width of the 90° rib before and after transverse stretching. (**a**) Thickness, (**b**) width.

Along the Path-Y path shown in Figure 16, we collect and compare the width and thickness of the 90° rib before and after transverse stretching, as shown in Figure 17.

As shown in Figure 17a, the thickness of the node before and after transverse stretching is basically unchanged, and the thickness of the transition zone and rib is slightly reduced. From Figure 17b, it can be seen that the width of the 90° rib has increased slightly. This is because the transverse tension is applied to the geogrid plate during the transverse stretching, and the material that has formed the longitudinal ribs is slightly deformed in the transverse direction.

By comparing the simulation results with the test data, the deviation can be calculated and the accuracy of the simulation method can be verified. The comparison between the simulation results of tensile forming and the test data is shown in Table 2.

It can be seen from Table 2 that the simulation results are basically consistent with the test, and the deviation is controlled within 10%. Therefore, the simulation method can be applied to the stretch forming analysis of the triaxial geogrids.

**Figure 18:**
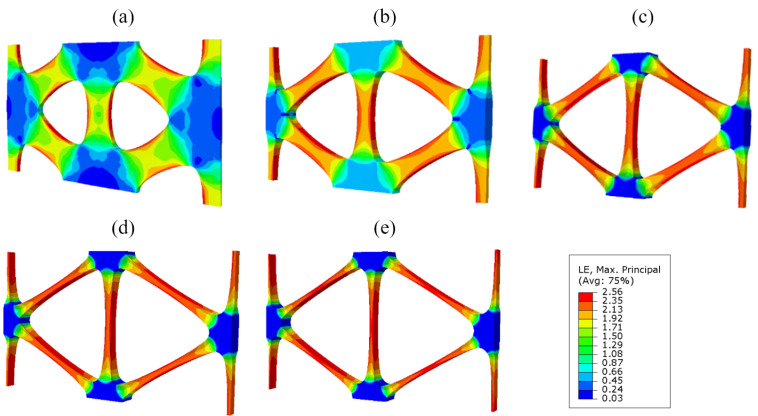
Simulation result of triaxial geogrid with different pre-punched diameters. (**a**) D = 2.0 mm, (**b**) D = 2.5 mm, (**c**) D = 3.0 mm, (**d**) D = 3.5 mm, (**e**) D = 4.0 mm.

Using this simulation method, the stretch process of triaxial geogrids with pre-punched diameters of 2.0 mm, 2.5 mm, 3.0 mm, and 4.0 mm were simulated. The stretching results are symmetrical along the X axis and the Y axis, and the obtained results are shown in Figure 18.

It can be seen from Figure 18 that when D = 2.0 mm, the nodes are completely destroyed during the stretching process, and regular-shaped nodes cannot be formed. When D = 2.5 mm, the nodes are partially destroyed, and the maximum thickness of the grid appears at the edge of the nodes. When D is no less than 3.0 mm, a regular and complete triaxial geogrid can be formed.

According to the analysis in Figure 8, the tensile strength of triaxial geogrid increases first and then decreases with the increase of the diameter of the pre-punched hole. When D is greater than 2.5 mm and less than 3 mm, the tensile strength of triaxial geogrid must have a maximum value; and when the tensile strength takes the maximum value, the nodes are not damaged after forming. The minimum pre-punched diameter of triaxial geogrid with complete and regular nodes can be obtained quickly and accurately by finite element simulation.

**Figure 19:**
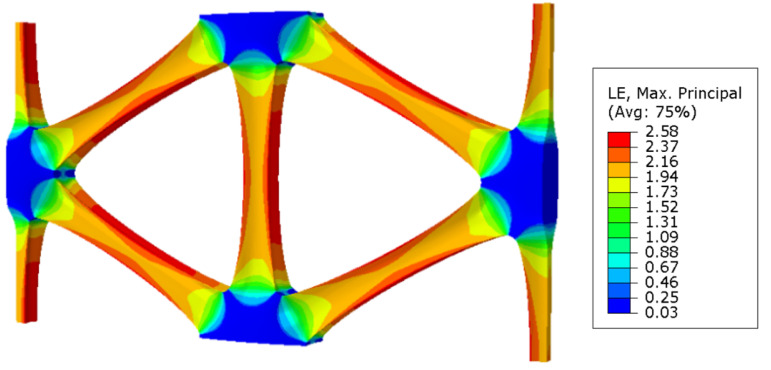
Simulation result of a triaxial geogrid with a pre-punched diameter of 2.6 mm.

For the pre-punched hole diameter D located in (2.5, 3.0) interval, with 0.1 mm as an interval, the numerical simulation of stretch forming was carried out with the same simulation method and process parameters from 2.6 mm to 2.9 mm. It was found that each sample could form a triaxial geogrid with regular node shape and complete structure after stretching. The simulation result for D = 2.6 mm is shown in Figure 19.

It can be seen from Figure 19 that under the test parameters, the nodes are not damaged after forming, and the minimum diameter D of the pre-punched hole is 2.6 mm. It can be further speculated that when D = 2.6 mm, the tensile strength of the triaxial geogrid can reach the maximum value.

When D = 2.0 mm, the node is completely destroyed and does not have the complete structure of the geogrid. When D = 2.5 mm, the morphology of the node in the pre-stretched area and the post-stretched area are different, therefore, data sampling for a geogrid with 2.5 mm pre-punched hole is not representative. Therefore, in this part, data sampling and analysis are mainly carried out respectively on the simulation results of stretch forming with pre-punched diameters of 2.6 mm, 3.0 mm, 3.5 mm, and 4.0 mm.

**Figure 20:**
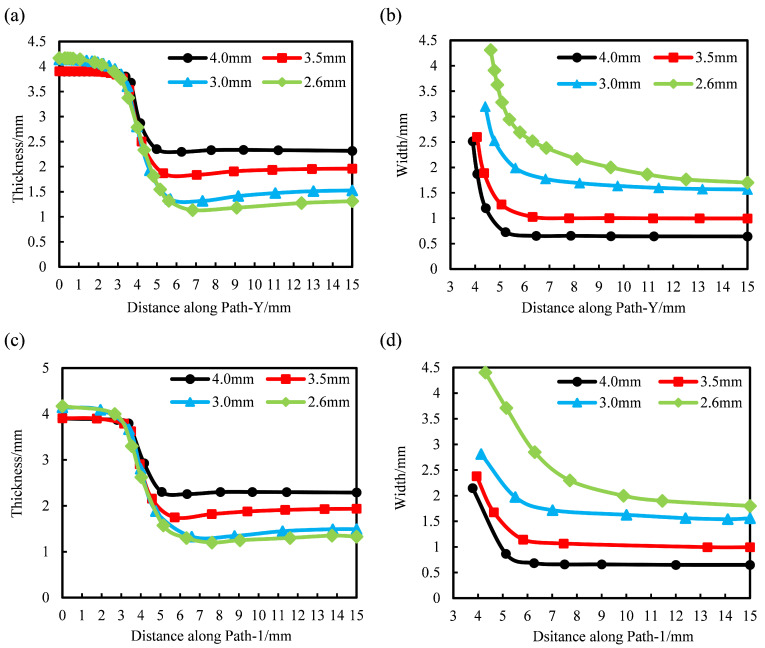
Variation of thickness and width of 90° bar along Path-Y and diagonal bar along Path-1. (**a**) Thickness of 90° bar along Path-Y, (**b**) width of 90° bar along Path-Y, (**c**) thickness of diagonal bar along Path-1, (**d**) width of diagonal bar along Path-1.

The thickness and width of industrial PP triaxial geogrids with different pre-punched diameters were measured along Path-Y and Path-1, shown in Figure 16. The distribution of the width and thickness of the 90° and diagonal bars when D = 2.6 mm, 3.0 mm, 3.5 mm, and 4.0 mm were obtained, which is shown in Figure 20.

Figure 20a,b represent the thickness and width distribution of the 90° bar along the Path-Y. Figure 20c,d represent the thickness and width distribution of the diagonal bar along the Path-1.

It can be seen from Figure 20a,b that on the premise that the nodes are not damaged, the thickness and width of the samples with the same pre-punched hole diameter show a decreasing trend along Path-Y. It can be seen from Figure 20a that the thickness of the nodes and ribs changes to a small extent, and the thickness of the transition zone changes drastically along Path-Y. It can be seen from Figure 20b that the width of the transition region varies greatly, and the width of the rib varies less. With the increase of the pre-punched hole diameter, the thickness of the node decreases slightly. The thickness of each sample in the transition zone is basically the same, while the width in the transition zone decreases significantly. The thickness of the rib increases, while the width of the rib decreases significantly.

When D is less than 3.0 mm, due to the large hole spacing before forming, more materials are involved in the stretching process, and the width of the rib after forming is relatively large; and the material in the central area of the rib shrinks greatly along the thickness direction, the degree of deformation in the thickness direction is large, so the width of the rib is greater than the thickness.

From Figure 20c,d, the variation rule of the width and thickness of the diagonal bar with the diameter of the pre-punched hole is the same as that of the 90° bar. Under the above conditions, when D = 2.6 mm, the thickness of the diagonal rib is the smallest and the width is the largest. When D = 4.0 mm, the thickness of the diagonal rib is the largest and the width is the smallest. The width and thickness of the diagonal bars with the same D are basically the same as those of the longitudinal bars. Therefore, when D is not less than 2.6 mm, the tensile strength and elongation at break of the longitudinal bars and the diagonal bars are basically the same and the mechanical performance is more balanced.

#### 3.3.2. Stress and Strain Analysis of Stretch Forming

The stress and strain of the stretch forming process were analyzed by Abaqus CAE, and the distribution law of the stress and strain was obtained. This section will analyze the distribution of stress and strain after the triaxial geogrid is stretched from two aspects: the distribution law of stress and strain along each path and the change law of stress and strain at special points.

**Figure 21:**
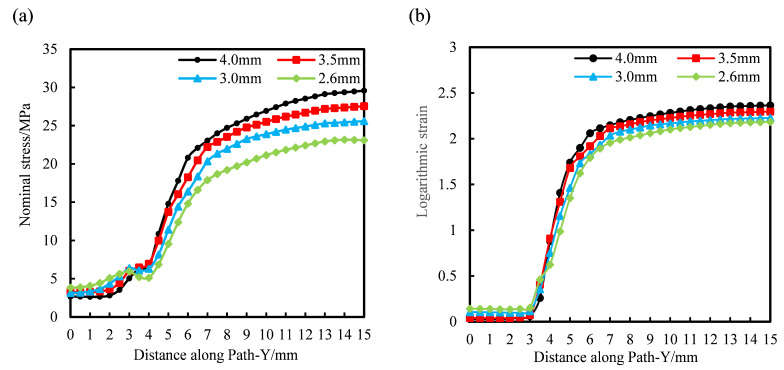

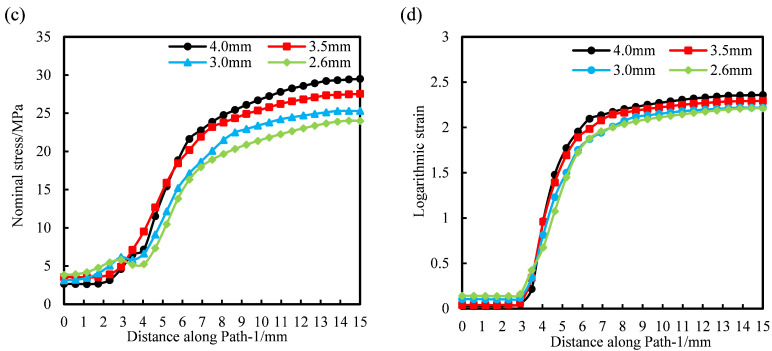
Variation law of stress and strain along Path-Y and Path-1 with the diameter of the pre-punched hole. (**a**) Nominal stress of 90° bar along Path-Y, (**b**) logarithmic strain of 90° bar along Path-Y, (**c**) nominal stress of the diagonal bar along Path-1, (**d**) logarithmic strain of the diagonal bar along Path-1.

Along Path-Y, starting from the starting point, data are collected at equal intervals. The data interval is 0.5 mm, and the values of stress and a logarithmic strain of 90° bar at each point are measured. The specific distribution law is shown in Figure 21a,b. In the same way, the distribution law of the stress and strain of the diagonal bar is measured along Path-1, as shown in Figure 21c,d.

It can be seen from Figure 21a that the internal stress of the node decreases with the increase of the diameter of the pre-punched hole, and the internal stress of the longitudinal rib increases with the increase of the diameter of the pre-punched hole. When D = 4.0 mm, the stress of longitudinal rib can reach 10 times that of the node. When D = 2.6 mm, the stress of longitudinal rib is 6 times that of the node, and the internal stress distribution is more uniform in this condition, so the tensile strength is higher in the room temperature tensile pulling off test.

It can be seen from Figure 21b that the strain distribution law is basically consistent with the stress distribution. The strain in the node region is small, and basically no deformation occurs during the stretching process. With the increase of the diameter of the pre-punched hole, the logarithmic strain of the node gradually decreases. The longitudinal rib has a large strain and is mainly involved in the deformation during the stretching process; and with the increase of the diameter of the pre-punched hole, the logarithmic strain of the longitudinal rib increases gradually.

The reason for the above phenomenon is that with the increase of the diameter of the pre-punched hole, the material participating in the deform of the rib decreases, and the rib can easily form stress concentration during the stretching process. However, when the diameter of the pre-punched hole is small, there are more materials involved in forming the rib, and the tensile force on the node increases during the stretching process, and the strain value of the node is large.

Figure 21c,d shows that the stress-strain distribution along Path-1 is basically the same as that of the 90° bar. It can be seen from Figure 21c that the diagonal bars are mainly involved in the deformation during the transverse stretching process, in which the node stress is small, and the rib stress is large. The internal stress of the diagonal rib can reach 6–10 times the internal stress of the node. It can be seen from Figure 21d that when D = 2.6 mm or 3.0 mm, the central strain of the diagonal rib is basically equal.

**Figure 22:**
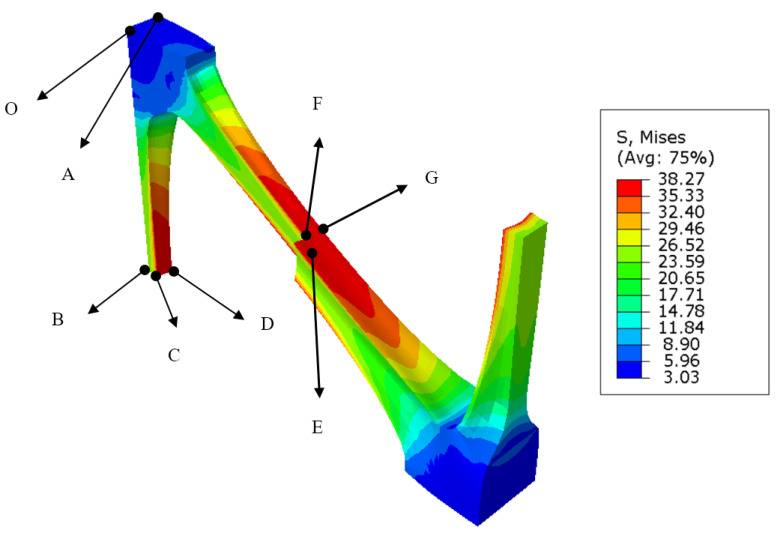
Stress-strain point position.

The internal stress and strain distribution of industrial PP triaxial geogrid can be measured along Path-Y and Path-1, but, as shown in Figure 15, the maximum stress and strain of triaxial geogrid appears on the surface of the rib. Therefore, it is necessary to select special points and measure their stress-strain values to obtain the overall stress-strain distribution law of triaxial geogrid. The surface center points, internal center points and edge center points of nodes, longitudinal ribs, and diagonal ribs are selected as representative points, which are shown in Figure 22.

In Figure 22, point O can represent the center of the node, point A represents the surface of the node. Point B is the inner center point of the longitudinal rib, point C represents the surface of the longitudinal rib, and point D represents the edge of the longitudinal rib. Point E represents the center point inside the diagonal rib, point F represents the surface of the diagonal rib, and point G represents the edge of the diagonal rib. We use σ to indicate stress, ϵ to indicate strain. We add a superscript after σ and ϵ, *U* indicates after longitudinal stretching, and *B* indicates after transverse stretching. We add a subscript after σ and ϵ, and the subscript indicates the selected points on the triaxial geogrid. For example, $\sigma_{O}^{U}$ represents the stress at point O after the longitudinal stretching is completed.

Table 3 shows the stress obtained at different positions of triaxial geogrid under the condition of different pre-punched hole diameters after longitudinal stretching.

Table 4 shows the stress obtained at different positions of triaxial geogrid under the condition of different pre-punched hole diameters after transverse stretching.

Table 5 shows the strain collected at special points of triaxial geogrid under different pre-punched hole diameters after longitudinal stretching.

Table 6 shows the strain collected at special points of triaxial geogrid under different pre-punched hole diameters after transverse stretching.

Combined with the data in Table 3, Table 4, Table 5 and Table 6, it can be found that, compared with before the transverse stretching, the changes of each point after the transverse stretching of triaxial geogrid are as follows: the stress at points O and A will increase, and the stresses at points B, E, F, and G will increase. The stress and strain at point C will increase, and the strain at point C will increase. As the diameter of the pre-punched hole increases, the strain at point O in triaxial geogrid after biaxial stretching will increase, the stress at point C will increase, the stress and strain values of A and F will increase, and the stress and strain at points B and E will decrease.

Therefore, transverse stretching will increase the stress value of the node, the inner center of the longitudinal rib, and each point of the diagonal rib. Moreover, transverse stretching will increase the strain value of the center and surface of the longitudinal rib and each point of the diagonal rib. When the diameter of the pre-punched hole increases, the stress on the node surface and longitudinal rib/diagonal rib surface of polypropylene triaxial geogrid will increase, and the stress at the center of longitudinal rib/diagonal rib will decrease; the strain on the center and surface of the node and the surface of the diagonal rib will increase, and the strain at the center of the longitudinal rib/diagonal rib will decrease.

## 4. Conclusions

In this paper, the relationship between the mechanical properties of industrial polypropylene triaxial geogrid and the diameters of pre-punched holes is studied by experiments combined with numerical simulation analysis, and the thickness, width, stress and strain distribution of triaxial geogrids with different diameters are obtained. The innovation of the paper is reflected in the analysis of pre-punched holes on the mechanical properties of triaxial geogrid through experiments and the application of finite element simulation to the forming process of triaxial geogrid. Furthermore, the feasibility of finite element simulation in the design of multi-axial geogrid is verified. The main conclusions are as follows.

1. When the diameter of the pre-punched hole is small, the node of triaxial geogrid will appear concave in the middle, the integrity of the node will be damaged, and fractures at the node will occur in the room temperature tensile test. When the diameter of the pre-punched hole is larger, the node will appear convex in the middle, and fractures in the transition zone often occur in the tensile fracture test at room temperature. The width of the triaxial geogrid rib decreases as the diameter of the pre-punched hole increases.

2. The tensile fracture tests at room temperature were carried out on the industrial polypropylene triaxial geogrid with a node center spacing of 30 mm, and it was found that the tensile strength increased first and then decreased with the increase of the diameter of the pre-punched holes, and the maximum point interval of the average tensile strength was obtained. Combined with numerical simulation analysis, it was obtained that when the pre-punched hole diameter is 2.6 mm, the comprehensive mechanical properties were the best. The fracture elongation of the rib in the 90° direction showed an overall decreasing trend with the increase of the diameter of the pre-punched hole, and the appearance of this phenomenon is related to the node concave in the middle.

3. The stress-strain curve at 373–413 K was obtained through the high-temperature tensile test of the 5A industrial polypropylene spline. After fitting in Abaqus, it was found that the Marlow constitutive model was applicable.

4. Through numerical simulation analysis of industrial polypropylene preheating process with different pre-punched hole diameters, it was found that a larger pre-punched hole was beneficial to heat transfer, so the heating was more sufficient. In addition, it was found that the temperature of each part was different under the same pre-punched condition, indicating that the subsequent stretching process was formed under a certain temperature gradient.

5. Through the simulation analysis of the industrial polypropylene stretching process, it was found that the transverse stretching had a certain influence on the geogrid after longitudinal stretching. In addition, the diameter of the pre-punched holes affected the shape of the nodes and ribs after forming, as well as the distribution of stress and strain.

The research is of great help to analyze the forming process of a triaxial geogrid, and is of great significance for regulating the mechanical properties of a triaxial geogrid. Therefore, it can promote the application of triaxial geogrid and the design of a new multi-axial geogrid.

## Figures and Tables

**Table 1:** Process parameter of stretching.

Parameter	Set Value
Material	Industrial PP
Stretching Ratio	3.0
Stretching Temperature	393 K
High Temperature Holding Time	5 min
Stretching Speed	100 mm/min
Thickness of Plates	4.0 mm

**Table 2:** Comparison of tensile forming simulation results with experimental data.

Items for Comparison	Simulation Results	Test Results	Deviation
Thickness of the node	3.91 mm	4.02 mm	−2.74%
Width of the node	5.82 mm	5.69 mm	+2.28%
Thickness of longitudinal rib	1.95 mm	1.94 mm	+0.52%
Width of longitudinal rib	0.99 mm	0.91 mm	+8.79%

**Table 3:** Stress at different positions of triaxial geogrid after longitudinal stretching. (MPa).

D	$\sigma_{O}^{U}$	$\sigma_{A}^{U}$	$\sigma_{B}^{U}$	$\sigma_{C}^{U}$	$\sigma_{D}^{U}$	$\sigma_{E}^{U}$	$\sigma_{F}^{U}$	$\sigma_{G}^{U}$
4.0 mm	2.27	2.39	29.38	36.51	35.89	18.65	22.26	21.86
3.5 mm	2.82	3.04	27.29	36.65	35.67	17.85	22.73	22.14
3.0 mm	4.37	4.92	25.57	37.59	36.77	16.16	21.91	21.45
2.6 mm	5.14	5.73	23.06	38.20	38.81	15.55	22.31	22.01

**Table 4:** Stress at different positions of triaxial geogrid after transverse stretching. (MPa).

D	$\sigma_{O}^{B}$	$\sigma_{A}^{B}$	$\sigma_{B}^{B}$	$\sigma_{C}^{B}$	$\sigma_{D}^{B}$	$\sigma_{E}^{B}$	$\sigma_{F}^{B}$	$\sigma_{G}^{B}$
4.0 mm	2.66	3.22	29.57	36.74	36.05	29.52	36.65	35.79
3.5 mm	3.26	3.87	27.55	36.95	35.89	27.51	37.02	35.86
3.0 mm	3.14	4.01	25.58	37.50	36.49	25.31	37.43	36.19
2.6 mm	3.82	4.61	23.08	38.22	38.08	24.00	38.48	35.56

**Table 5:** Logarithmic strain at different positions of triaxial geogrid after longitudinal stretching.

D	$\epsilon_{O}^{U}$	$\epsilon_{A}^{U}$	$\epsilon_{B}^{U}$	$\epsilon_{C}^{U}$	$\epsilon_{D}^{U}$	$\epsilon_{E}^{U}$	$\epsilon_{F}^{U}$	$\epsilon_{G}^{U}$
4.0 mm	0.034	0.033	2.359	2.534	2.517	1.944	2.105	2.090
3.5 mm	0.040	0.039	2.287	2.528	2.502	1.894	2.118	2.097
3.0 mm	0.071	0.076	2.224	2.542	2.521	1.776	2.083	2.058
2.6 mm	0.102	0.133	2.182	2.631	2.633	1.694	2.124	2.123

**Table 6:** Logarithmic strain at different positions of triaxial geogrid after transverse stretching.

D	$\epsilon_{O}^{B}$	$\epsilon_{A}^{B}$	$\epsilon_{B}^{B}$	$\epsilon_{C}^{B}$	$\epsilon_{D}^{B}$	$\epsilon_{E}^{B}$	$\epsilon_{F}^{B}$	$\epsilon_{G}^{B}$
4.0 mm	0.028	0.032	2.365	2.540	2.521	2.359	2.542	2.523
3.5 mm	0.040	0.047	2.297	2.537	2.509	2.293	2.544	2.519
3.0 mm	0.104	0.129	2.227	2.542	2.515	2.218	2.549	2.518
2.6 mm	0.141	0.180	2.184	2.634	2.609	2.211	2.628	2.554

## Data Availability

The data used to support the findings of this study are available from the corresponding author upon request.
